# Necrotizing Retinitis Secondary to Congenital Cytomegalovirus Infection Associated with Severe Combined Immunodeficiency

**DOI:** 10.1155/2016/1495639

**Published:** 2016-11-23

**Authors:** Pehmen Yasin Ozcan, Hasan Tolga Celik, Kenan Sonmez, Melda Celik

**Affiliations:** ^1^Ministry of Health Sanliurfa Education and Research Hospital, Sanliurfa, Turkey; ^2^Neonatal Intensive Care Unit, Department of Neonatology, Sanliurfa Children's Hospital, Sanliurfa, Turkey; ^3^Ministry of Health Ankara Ulucanlar Eye Training and Research Hospital, Ophthalmology Department, Ankara, Turkey; ^4^Sanliurfa Children's Hospital, Sanliurfa, Turkey

## Abstract

A 20-day-old male infant who was born at 39 weeks of gestation was admitted to neonatal intensive care unit due to severe respiratory insufficiency. In retinal examination, peripheric retinal white-black color areas that correspond to necrotizing retinitis, moderate vitritis, macular and optic nerve head involvement, vascular leakage, and sheathing indicating perivasculitis were revealed. Despite the fact that CMV specific IgM was undetectable, CMV DNA with high viral load was found in his blood sample by means of real-time polymerase chain reaction assay. Serologic examination (IgM) for rubella, toxoplasma, herpes simplex type 2, and human immunodeficiency virus (anti-HIV) was negative. During the further evaluation for systemic immune dysfunction, decreased immunoglobulin and lymphocyte levels that confirm the diagnosis of severe combined immunodeficiency have been reached. Although given systemic intravenous ganciclovir and antibiotics treatment, the patient died at the 4th month of life due to respiratory insufficiency.

## 1. Introduction

Cytomegalovirus (CMV) is the most common cause of congenital and perinatal viral infections over the world. Incidence of congenital human CMV ranges from 0.3 to 1.0% of all live births, much as 10% of congenitally infected babies have indications of infection at birth [1–4]. Although most of neonates with congenital CMV infection are asymptomatic in the early period of life, some of them could develop neurologic complications such as microcephaly, hydrocephalus, mental retardation, hearing disorder, chorioretinitis, and rarely finger anomalies [5]. CMV retinitis in children may be seen in immunocompromising conditions including severe combined immunodeficiency syndrome (SCID) and acquired immunodeficiency syndrome after bone marrow or renal transplantation and chemotherapy.

Here, we reported an unusual case which comprises a congenital necrotizing cytomegalovirus retinitis associated with severe combined immunodeficiency

## 2. Case Report

A male infant who was the second gestation of a 25-year-old healthy mother was born by vaginal delivery subsequent to 39 weeks of gestation. There was no consanguinity between the parents. Initially, the baby was fed with only breast milk. On the 20th day of his life he was admitted to our neonatal intensive care unit due to respiratory distress. In the physical examination tachypnea, intercostal retractions and crackles were observed. As diffuse interstitial pneumonic infiltration had been shown on the chest radiography, empiric antibiotic treatment (ampicillin, gentamicin) was initiated. Neither bacterial growth on the blood culture nor history of maternal infection during the pregnancy period was detected. Serological tests were negative for CMV, rubella, toxoplasma, herpes simplex type 2, and human immunodeficiency virus. Ventriculomegaly and periventricular calcifications were determined on transfontanellar ultrasound and computerized tomography.

**Figure 1:**
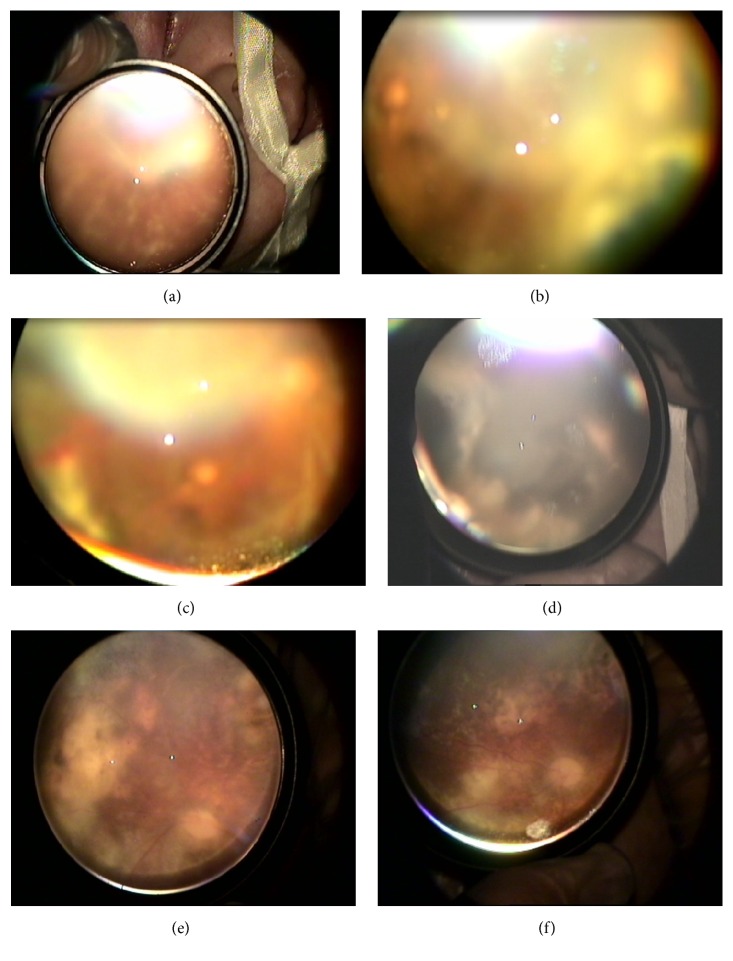
Color fundus images of the eye. (a) Periphlebitis and vascular sheathing, (b) peripheral retinal necrosis and foveal atrophy, (c) moderate vitritis, retinal hemorrhage, and necrotizing retinitis, (d) white-black colored, diffuse necrotizing retinitis, and (e), (f) after ganciclovir treatment, resolved signs of moderate vitritis, vascular sheathing and periphlebitis, and existing of paled optic disc.

Ophthalmic examination was performed with indirect biomicroscopy and +30 D funduscopic lens. Intraocular pressures of both eyes were 16 mmHg with tono-pen contact tonometer. Retinal examination revealed that bilateral moderate vitritis, dense pigmented, white-black colored diffuse scars involving all quadrants of the periphery retina corresponding to the necrotizing retinitis, as well as perivasculitis, vascular sheathing, retinal hemorrhages, and even resembling frosted branch angiitis were seen around posterior pole (Figures 1(a), 1(b), 1(c), and 1(d)).

Because of having suspicious retinal and clinical findings of any viral infection, particularly congenital CMV infection, further evaluation was performed. Although CMV specific IgM was undetectable, high blood CMV viral load (CMV DNA, 7200000 copy/mL) was found in his blood sample with the real-time polymerase chain reaction assay. Systemic intravenous ganciclovir (10 mg/kg/day) treatment was initiated for 15 days. After the treatment, retinal findings including vascular sheathing and leakage and perivasculitis were resolved along with persisting necrotized peripheral retina (Figures 1(e) and 1(f)). The general health condition of patient was unresponsive to the ongoing antibiotic and antiviral administrations. In blood analyses performed for investigating immune dysfunction, decreased count of total lymphocytes, lymphocyte subgroups, and immunoglobulins levels that indicate the diagnosis of severe combined immune dysfunction were gained. In spite of all medical interventions, the patient died during the 4th month of life due to severe respiratory insufficiency.

## 3. Discussion

Nearly 10% of vertically CMV infected newborns present or develop severe signs of cytomegalic inclusion disease (CID) that contains the classical triad including chorioretinitis, microcephaly, and cerebral calcifications [1–5]. The clinical features of our patient corresponded to the triad of congenital CMV infections and serum CMV DNA was also positive.

Vision loss in congenital CMV infection is caused by chorioretinitis, optic neuropathy, and cortical vision loss [6]. The incidence of cytomegalovirus (CMV) retinitis has been reported to be up to 25% of infants with severely symptomatic congenital cytomegalovirus [2]. CMV retinitis appears as a retinitis composed of perivascular areas of retinal necrosis with intraretinal hemorrhages, vasculitis, cotton-wool spots, and mild vitritis [7]. Ophthalmologic examination in our patient revealed bilateral perivasculitis, active proliferative vitreoretinopathy formation, moderate vitritis, and dense pigmented white-black colored diffuse necrotizing retinitis that resemble chorioretinal scar formation involving all quadrants of the retina. The retinitis in congenitally infected newborns differs from that described in immunocompromised subjects, since it usually does not progress after birth if the child is otherwise immunocompetent [8]. In immunocompetent newborns with CMV retinitis, mild chorioretinal scarring to retinal necrosis like in our patient occurs in up to 20% of symptomatic neonates. The late diagnosis of systemic immune dysfunction in our patient following established chorioretinal scar formation and retinal necrosis indicates the lack of newborn screening programme for SCID in our state.

Exact treatment for congenital CMV-associated retinitis has not been well established [9]. Based on current evidences of treatment, ganciclovir (GCV) administration would seem appropriate to recommend early treatment of retinitis; that is why we have decided to prescribe the currently recommended therapeutic regimens for GCV. After 2 weeks to the ganciclovir treatment apart from chorioretinal scar formation, retinal findings were resolved and vitritis was abated also. Unfortunately, the patient died at the 4th month of life due to respiratory insufficiency.

This case supports that the absence of CMV IgG antibody does not eliminate the possibility of an CMV infection. The presence of a very high viral load > 100,000 copies/mL should always trigger an eye examination, since high CMV viral loads are a risk factor for CMV retinitis and infants with SCID are at risk for severe life threatening and sight threatening CMV infections, as illustrated in this patient.
